# Division of Labor, Bet Hedging, and the Evolution of Mixed Biofilm Investment Strategies

**DOI:** 10.1128/mBio.00672-17

**Published:** 2017-08-08

**Authors:** Nick Vallespir Lowery, Luke McNally, William C. Ratcliff, Sam P. Brown

**Affiliations:** a Institute of Evolutionary Biology, School of the Biological Sciences, University of Edinburgh, Edinburgh, United Kingdom; b Centre for Immunity, Infection and Evolution, School of the Biological Sciences, University of Edinburgh, Edinburgh, United Kingdom; c School of Biology, Georgia Institute of Technology, Atlanta, Georgia; University of Washington

**Keywords:** biofilms, evolution, microbial ecology

## Abstract

Bacterial cells, like many other organisms, face a tradeoff between longevity and fecundity. Planktonic cells are fast growing and fragile, while biofilm cells are often slower growing but stress resistant. Here we ask why bacterial lineages invest simultaneously in both fast- and slow-growing types. We develop a population dynamic model of lineage expansion across a patchy environment and find that mixed investment is favored across a broad range of environmental conditions, even when transmission is entirely via biofilm cells. This mixed strategy is favored because of a division of labor where exponentially dividing planktonic cells can act as an engine for the production of future biofilm cells, which grow more slowly. We use experimental evolution to test our predictions and show that phenotypic heterogeneity is persistent even under selection for purely planktonic or purely biofilm transmission. Furthermore, simulations suggest that maintenance of a biofilm subpopulation serves as a cost-effective hedge against environmental uncertainty, which is also consistent with our experimental findings.

## IMPORTANCE

Cell types specialized for survival have been observed and described within clonal bacterial populations for decades, but why are these specialists continually produced under benign conditions when such investment comes at a high reproductive cost? Conversely, when survival becomes an imperative, does it ever benefit the population to maintain a pool of rapidly growing but vulnerable planktonic cells? Using a combination of mathematical modeling, simulations, and experiments, we find that mixed investment strategies are favored over a broad range of environmental conditions and rely on a division of labor between cell types, where reproductive specialists amplify survival specialists, which can be transmitted through the environment with a limited mortality rate. We also show that survival specialists benefit rapidly growing populations by serving as a hedge against unpredictable changes in the environment. These results help to clarify the general evolutionary and ecological forces that can generate and maintain diverse subtypes within clonal bacterial populations.

## INTRODUCTION

After billions of years of evolution, many organisms retain an impressive capacity for innovation and adaptation to their environment. However, for core traits such as durability and the reproductive rate, improvements in one will often come at the cost of another—indeed, understanding how adaptation occurs when key fitness parameters are constrained by tradeoffs lies at the core of life history theory (1). While most life history theory has been developed with large multicellular organisms in mind, microbes also exhibit classical trade-offs in fecundity and longevity, with faster-growing lineages tending to be more fragile (2, 3). Understanding how microbes manage such trade-offs remains a major goal in microbiology from both mechanistic (4) and ecological (5) perspectives.

Multiple mechanisms for enhancing durability and longevity are available to microbes but typically come at the cost of reduced metabolic proficiency. Spore formation is perhaps the clearest example of a high-survival, low-fecundity phenotype; by encasing the genome and some essential metabolic machinery in a thick and extremely resistant cell wall, dormant spores can survive for extraordinarily long durations (6, 7). Alternatively, cells may form metabolically dormant persister cells capable of surviving diverse environmental insults (8, 9). Finally, many microbial species form biofilms where dense cell packing and production of a protective extracellular matrix provide broad resistance to stressors such as desiccation, predation, or chemical insult (10) but also limit space and nutrient diffusion, thereby reducing growth rates.

Clonally reproducing microbes present an interesting and experimentally tractable system in which to examine mixed behavioral strategies. Across many species of microbes, single genotypes can produce coexisting subpopulations of rapidly dividing planktonic cells and slow-growing or dormant stress-tolerant phenotypes, but focus is often given to a specific phenotype of interest rather than the balance between alternate phenotypes. In this study, we examined how the trade-off between the survival and growth of individual cells drives the evolution of mixed biofilm/planktonic investments on a lineage scale under diverse environmental conditions. Specifically, we build population dynamic models of bacteria in patchy environments where cells can switch between biofilm and planktonic states within ephemeral patches (via planktonic colonization of the biofilm and dispersal from the biofilm to the planktonic state) and can also be transmitted among patches as either biofilm or planktonic cells. We then ask under what conditions investment in biofilm is favored, given that biofilms grow more slowly. If only one phenotype (i.e., biofilm or planktonic) is favored for transmission to a new patch, does it ever pay to diversify into the cell type that is, from a transmission perspective, a dead end? Our model predicts that phenotypic diversification can pay across a wide range of environmental conditions, as rapidly growing planktonic cells can function as a “growth engine” providing higher levels of future planktonic and biofilm cells for transmission. We then test our model predictions by using stochastic simulations and experimental evolution of biofilm allocation in the environmental microbe *Pseudomonas aeruginosa*.

## RESULTS

### Biofilm growth dynamics.

**FIG 1:**
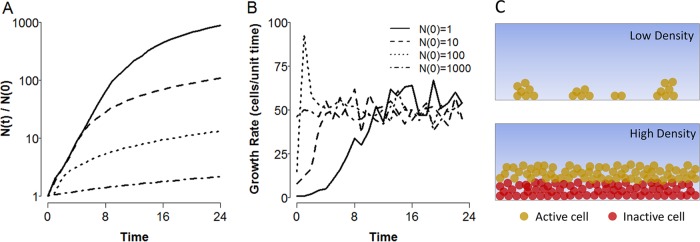
Biofilms grow subexponentially. (A, B) Numbers of accumulated cells (A) and lineage growth rates (B) through time for agent-based simulations of a 2D biofilm growing on a 1D surface. Various inocula (see inset) were allowed to grow for a fixed time period under nutrient-rich conditions. Simulations were implemented with the agent-based simulation platform iDynoMiCS (13). (C) Schematic depiction of nutrient depletion leading to growth arrest in the biofilm interior. Diffusion and active consumption of nutrients in the outermost layers of the biofilm (yellow cells) result in starvation conditions for cells in the interior regions (red cells).

While it is well known that planktonic cells accumulate exponentially in nutrient-rich environments, it is less clear whether close-packed biofilm cells would follow the same functional form (note that here we are considering biofilm growth in the absence of any coupling with the planktonic compartment, i.e., no cells dispersing from the biofilm or colonizing from the bulk). We hypothesize that sparse colonization allows for lineages to grow exponentially, as there is little steric inhibition or nutrient depletion to slow growth. However, once confluence across the surface is reached, further growth is restricted to a fixed depth within the outermost layer in biofilms because of space and diffusion limitations (11, 12). We explore this conjecture by using the individual-based simulation platform iDynoMiCS (13) to simulate a simple two-dimensional (2D) biofilm and find that after an initial period of exponential growth, cell accumulation decays to a linear function in time (Fig. 1). More generally, we find that the rate of biofilm growth depends on the geometry of the system being considered (see Text S1 in the supplemental material), with the growth rate following a polynomial of order equal to the dimensionality of the system (i.e., for a 3D sphere, biofilm cells accumulate as a cubic in time). However, it should be noted that for finite volumes, there is a constant downward pressure through time on the order of the growth polynomial as the biofilm reaches confluence across each dimension (e.g., initially cubic expansion in three dimensions will decay to quadratic expansion in two dimensions once the limit in the *z* direction is reached). These findings highlight that while biofilm cells do not face the extreme growth penalty of resistant spores or persister cells, they face a significant and compounding growth deficit in comparison with the exponential growth of planktonic populations.

10.1128/mBio.00672-17.1 TEXT S1  Geometry and biofilm growth. Download TEXT S1, PDF file, 0.5 MB. Copyright © 2017 Lowery et al. 2017 Lowery et al. This content is distributed under the terms of the Creative Commons Attribution 4.0 International license.

### Coupled biofilm-plankton dynamics.

We next model a growing bacterial microcosm within which cells grow in one of two compartments, the planktonic (P) phase within the bulk fluid and the biofilm (B) phase attached to a surface and in contact with the bulk. The compartments are coupled such that biofilm cells can disperse to the planktonic phase and planktonic cells can colonize the biofilm. Cells in each compartment also divide, with planktonic cells growing exponentially and biofilm cells growing linearly (i.e., we assume a finite 2D surface available for biofilm colonization and ignore the initial superlinear growth period).

With the biofilm limited to linear expansion, we reasoned that the effects of growth within and dispersal from the biofilm would be negligible when coupled to exponential growth in the planktonic phase. This simplification was shown to be reasonable by comparing numerical simulations of the full model and a simplified model with no biofilm growth or dispersal (Fig. S1), yielding the model system outlined in equations 1 to 4 for within-patch growth. By setting the growth of the biofilm to zero, this simplified framework renders biofilm cells functionally equivalent to spores or persisters as described above, i.e., a subpopulation of nondividing cells supported by the growth of vegetative cells, which presumably must provide some other benefit (e.g., environmental resistance) to the overall population to counteract this loss of fitness or else be lost from the population.

10.1128/mBio.00672-17.4 FIG S1  Comparison of full and simplified models. Numerical results for within-patch models with quadratic (*dB*/*dt* = *g_quadratic_B*^1/2^ + *cP* − *dB*), linear (*dB*/*dt* = *g*_*linear*_ + *cP* − *dB*), and no (*dB*/*dt* = *cP*) biofilm growth. In all cases, *t* = 50, *B_0_* = 0, *P_0_* = 5,000,000, and 0.02 ≤ *r* ≤ 0.12 (blue-red color scale). For the models featuring biofilm growth, *g*_*linear*_ = 40,000, *g*_*quadratic*_ = 1,000, and *d* = 0.01 in both cases; the reduced model with no biofilm growth sets *d* = *g* = 0. Overall dynamics are similar. In general, increasing biofilm growth rates (i) restrict the instances of the maximum in biofilm cells occurring at intermediate colonization rates to higher planktonic growth rates and (ii) reduce the differences in biofilm cell accumulation across growth rates. Download FIG S1, PDF file, 0.1 MB. Copyright © 2017 Lowery et al. 2017 Lowery et al. This content is distributed under the terms of the Creative Commons Attribution 4.0 International license.

Our simplified model framework results in the following coupled differential equations:

(1) $$\frac{dP}{dt}=\left(r-c\right)P$$

(2) $$\frac{dB}{dt}=cP$$

where *B* and *P* are the numbers of biofilm and planktonic cells, respectively, *r* is the exponential growth rate, and *c* is the rate of colonization of the biofilm (with 0 ≤ *c* < *r*). Note that, in general, *c* need not be bounded by *r* and arbitrarily high values of *c* would result in a decline in *P* as switching to the biofilm phase outpaces planktonic growth, giving a sharp trade-off between the two compartments; we discuss this case in the context in which it arises below. Solving equations 1 and 2 as a function of time yields our within-patch population model as follows:

(3) $$P\left(t\right)=P_{0}e^{(r-c)t}$$

(4) $$B\left(t\right)=\frac{c}{r-c}[P\left(t\right)-P_{0}]$$

where *P_0_* is the planktonic inoculum.

**FIG 2:**
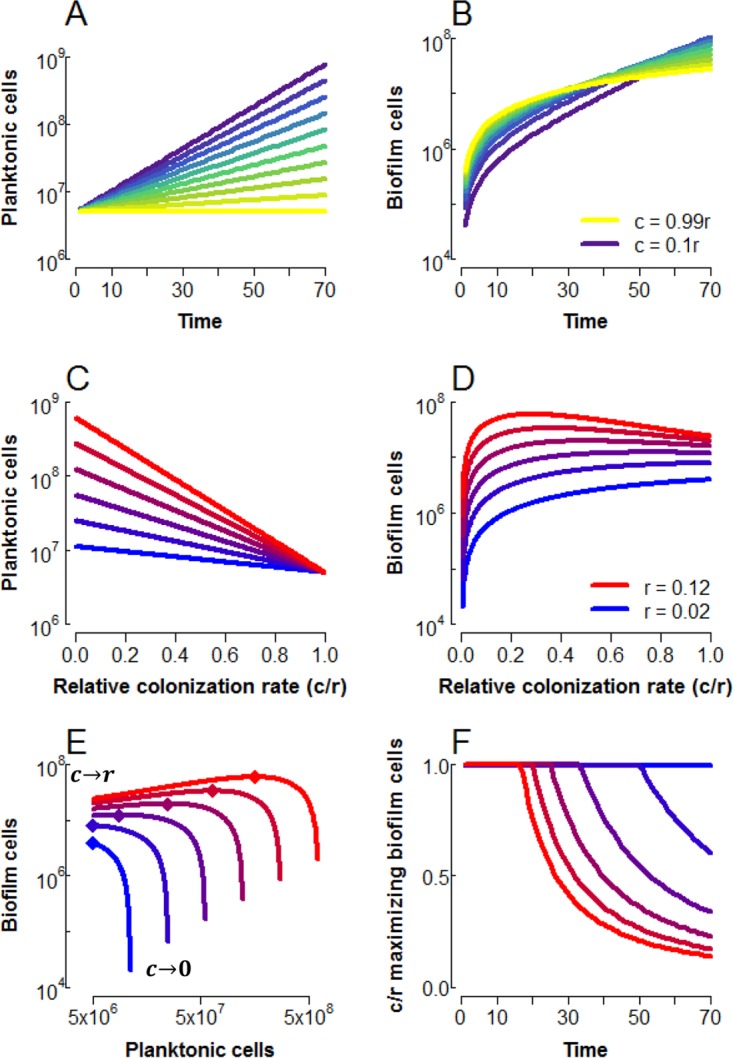
Maximal biofilm can be driven by planktonic growth. (A, B) Time series of planktonic (A) and biofilm (B) cells for colonization rates (*c* values) between 0.1 *r* (purple) and 0.99 *r* (yellow), with *r* = 0.08, *P_0_* = 5,000,000. (C, D) Planktonic (C) and biofilm (D) cells as a function of colonization rate relative to growth rate for *t* = 40, 0.02 ≤ *r* ≤ 0.12 (blue-red color scale), *P_0_* = 5,000,000. (E) Biofilm cells plotted against planktonic cells under the same conditions as in panels C and D. The colonization rate varies over each curve, with the endpoints indicated by labels (the relative colonization rate, *c/r*, approaches 1 at the left and zero at the right). Diamonds indicate the maximum in biofilm cells. (F) Relative colonization rate, *c/r*, at which biofilm is maximized. Note that in panels D and E, the limit of *c* = 0 is omitted, as this prevents any formation of biofilm cells.

The within-patch model reveals a temporal trade-off in biofilm accumulation with increasing colonization (Fig. 2). As expected, planktonic cells decline monotonically with an increasing colonization rate, *c*, as more cells are siphoned from the planktonic to the biofilm compartment (Fig. 2A and C). The biofilm, however, shows more interesting dynamics with changing rates of colonization (Fig. 2B and D). At *c* = 0, no biofilm cells accumulate, and as *c* approaches *r*, all new planktonic cells colonize the biofilm, resulting in a static planktonic population and linear accumulation of biofilm cells (Fig. 2A and B, yellow lines). However, when the planktonic fraction is allowed to expand exponentially (with 0 < *c* < *r*), the biofilm also accumulates cells roughly exponentially [once *P*(*t*) >> *P_0_*] at a constant fraction *c*/(*r*^−^ *c*) of the planktonic population. High colonization rates thus provide more biofilm cells at short time scales, while lower colonization rates maximize biofilm over longer periods of growth (Fig. 2B).

As shown in Fig. 2D, we find that the colonization rate maximizing biofilm cells declines with increasing planktonic growth rates, giving a humped shape in biofilm as a function of the colonization rate. We can find an analytical condition for this relationship by examining the slope of *B* as a function of *c* as *c* approaches *r* (see Text S2 for derivation) as follows:

(5) $$\underset{c\to r}{\lim}\left(\frac{dB}{dc}\right)=-\frac{1}{2}P_{0}t\left(rt-2\right)$$

If the slope is negative, this would imply an interior maximum in *B* at some value of *c* that is <*r* (as biofilm cells necessarily increase as *c* increases from zero). We find that this limit is negative when the product of *rt* is >2; i.e., the presence of a humped relationship in *B* requires patch quality (the product of *rt*) to exceed a minimal threshold value.

10.1128/mBio.00672-17.2 TEXT S2  Derivation of equation 5. Download TEXT S2, PDF file, 0.5 MB. Copyright © 2017 Lowery et al. 2017 Lowery et al. This content is distributed under the terms of the Creative Commons Attribution 4.0 International license.

These results suggest that the colonization rate maximizing biofilm will depend on opportunities for growth in the planktonic state (governed by growth rate *r* and growth duration *t*), which we explore further in Fig. 2E and F. In Fig. 2E, plotting biofilm cells against planktonic cells reveals that while limited growth (blue lines) leads to an allocation trade-off (i.e., increasing *B* necessarily comes at the cost of decreasing *P*), an increasing growth rate decouples this trade-off, with *B* maximized at diminishing colonization rates *c*, depicted explicitly in Fig. 2F. However, as colonization decreases beyond this point, biofilm declines sharply as colonization tends to zero.

Under high growth regimes, the planktonic fraction may therefore be viewed as a “growth engine” to maximize biofilm; when within-patch conditions are sufficiently favorable to planktonic division (equation 5), reducing the biofilm colonization rate, *c*, below the maximum increases the net flux of cells into the biofilm by expanding the pool of dividing planktonic cells, *P*. This growth engine effect is sufficient to drive colonization rates maximizing biofilm down to a fraction of the growth rate (Fig. 2F).

### Evolutionary model.

While intermediate colonization rates may maximize biofilm, we note that any allocation toward the biofilm comes at the cost of total population size (as shown in equation 6)

(6) $$\frac{d[P(t)+B(t)]}{dc}=-\frac{rP_{0}\{1+[(r-c)t-1]e^{(r-c)t}\}}{{\left(r-c\right)}^{2}}$$

which is strictly negative when *t* is >0 and equal to 0 when *t* equals 0. Given this trade-off between the biofilm and total population size, what conditions would favor biofilm investment (*c* of >0)? We can examine the evolutionary consequences of allocation in the within-patch population model by constructing a life cycle in which a population colonizes successive patches through space and/or time (i.e., migration between patches or remaining in a single patch that experiences periodic disturbances). We define a fitness function by assigning transmission probabilities *k*_*p*_ and *k*_*b*_ that a given cell from the respective planktonic or biofilm compartment will go on to found a new patch as follows:

(7) $$W=k_{p}P\left(t\right)+k_{b}B\left(t\right)$$

or, explicitly, per founding cell (analogous to the reproductive number “R0” framework common to parasite epidemiology and evolution [14]) as follows:

(8) $$W\left(c,r,t\right)=k_{p}e^{\left(r-c\right)t}+k_{b}[e^{(r-c)t}-1]\frac{c}{r-c}$$

**FIG 3:**
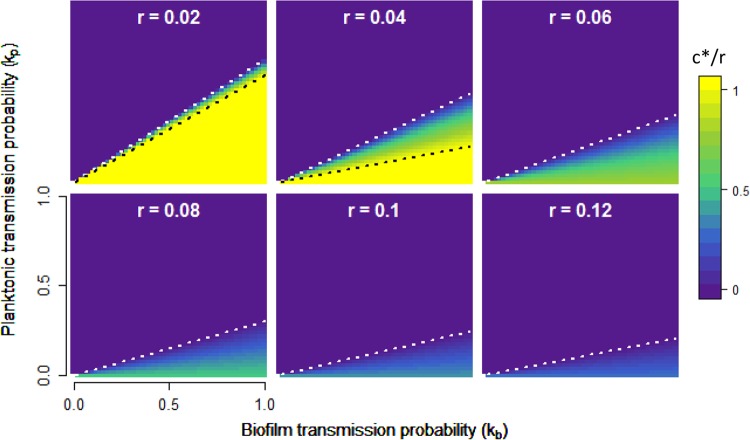
Optimal colonization rate, c*, as a function of the reproductive value of planktonic and biofilm cells. Contour plots show the relative colonization rate (c*/*r*, yellow-purple color scale) optimizing fitness (equation 8). Each panel displays c*/*r* as a function of *k*_*p*_ and *k*_*b*_. Across panels, the growth rate, *r*, increases from 0.02 to 0.12, with *t* = 40 in all cases; similar plots varying *t* are displayed in Fig. S2. A white dotted line indicates the point at which c* transitions from 0 to >0, while a black dotted line indicates the threshold at which c* becomes 1 (see text and Text S3).

Equation 8 allows us to interrogate the fitness consequences of biofilm investment strategies (colonization rate *c*) across a wide array of ecological parameters. *k*_*p*_ and *k*_*b*_ capture the reproductive value of each cell type and can be interpreted equivalently as a per-cell transmission probability or as the fraction of each subpopulation able to be transmitted successfully; they will dictate how well a given cell type (biofilm or planktonic) can survive the interpatch transition and be influenced by the nature of the environment. Growth time *t* describes the disturbance regime, i.e., how long a population can stay in a single patch, and *r* measures the nutrient quality of individual patches or how rapidly planktonic cells can divide within the patch. We define c* as the optimal colonization rate maximizing fitness *W* under a given ecological condition and display the behavior of c* as a function of transmission parameters *k*_*p*_ and *k*_*b*_ in Fig. 3.

There are three general strategies microbes may adopt in maximizing fitness, devoting all resources to the biofilm fraction (c* = *r*, below black dotted lines in Fig. 3), splitting resources between the two fractions (0 < c* < *r*, between dotted lines in Fig. 3), and devoting all resources to the plankton (c* = 0, above white dotted lines in Fig. 3). In Text S3, we investigate the conditions governing the two transitions defining these regimes by examining the behavior of equation 8 in more detail.

10.1128/mBio.00672-17.3 TEXT S3  Derivation of coexistence thresholds (Fig. 3). Download TEXT S3, PDF file, 0.5 MB. Copyright © 2017 Lowery et al. 2017 Lowery et al. This content is distributed under the terms of the Creative Commons Attribution 4.0 International license.

### Trade-offs in allocation.

When growth opportunities are limited (Fig. 3, upper left panel), we see evidence of a sharp tradeoff between *B* and *P* investment, with very little parameter space allowing intermediate investments (0 < c* < *r*). In the limit of zero within-patch growth, the allocation decision becomes a zero-sum game, with no allowance for intermediate investment, as follows:

(9) $$\frac{dW}{dc}{|}_{r=0}={te}^{-ct}\left(k_{b}-k_{p}\right)$$

In this no-growth scenario (*r* = 0), if biofilm cells have greater transmission value (*k*_*b*_ > *k*_*p*_), then total biofilm investment is favored; if not, then total planktonic cell investment is favored, giving a strict trade-off defined by whichever fraction is preferentially transmitted between patches.

This simple zero-sum logic is intuitive but fails significantly under more permissive growth conditions (i.e., increasing *r* and/or *t*; Fig. 3; Fig. S2), where we see an intermediate level of colonization is favored over a relatively large portion of the parameter space. Despite large transmission advantages to biofilm cells, the intermediate colonization regime extends to the boundary of *k*_*p*_ = 0 (black dashed line undefined for *r* > 0.04, Fig. 3), such that even when planktonic cells have zero probability of founding a new patch, the vast majority of the population is still allocated to that fraction. This result follows from the dynamics of the within-patch model (equations 3 and 4; Fig. 2); when *k*_*p*_ = 0, fitness is determined entirely by the size of the biofilm population, which is maximized at low colonization rates under conditions favoring growth because of the driving force of the planktonic growth engine.

10.1128/mBio.00672-17.5 FIG S2  Optimal colonization rate, c*, as a function of reproductive value for planktonic and biofilm cells. c*/*r* is plotted under the same conditions as in Fig. 3, but panels vary the growth period as opposed to the growth rate (here *r* = 0.08), increasing from left (*t* = 10) to right (*t* = 70). Download FIG S2, PDF file, 0.1 MB. Copyright © 2017 Lowery et al. 2017 Lowery et al. This content is distributed under the terms of the Creative Commons Attribution 4.0 International license.

### Biofilm as a hedge against environmental instability.

In our evolutionary model (Fig. 3), we assume that lineages can adapt their allocation decision making (c*) in the context of constant transmission weightings *k*_*p*_ and *k*_*b*_. However, the relative success of biofilm versus planktonic cell propagules is likely to vary extensively in time as a function of unpredictable biotic and abiotic stresses (i.e., changing *k*_*p*_ relative to *k*_*b*_). Despite reduced colonization rates leading to larger planktonic populations, sharply diminishing returns from further decreases in *c* (Fig. 2E) suggest that the biofilm compartment has the potential to function as a cost-effective hedge against unpredictable selective events. At all growth rates, populations can exchange a small reduction in the size of the planktonic population for a massive increase in the biofilm population by raising the rate of colonization from a minimal value; returning to equations 1 and 2, taking the ratio of the biofilm to the planktonic growth rate yields the fraction *c*/(*r* − *c*), which approaches infinity as *c* approaches *r*.

**FIG 4:**
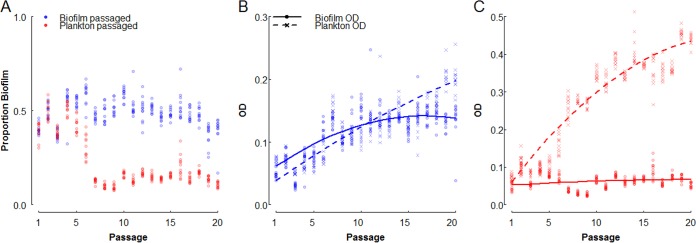
Selection experiments recapitulate model predictions. *P. aeruginosa* PAO1 was grown at room temperature in 96-well plates each well of which contained a glass bead, and every 12 h, populations were fractionated, measured, and passaged to a new well. Shown are the proportion of biofilm (defined as the ratio of the biofilm OD to the sum of the ODs of the two fractions) (A) and fractionated ODs of biofilm growth-selected (*k*_*b*_ = 1, *k*_*p*_ = 0) (B) and planktonic-growth-selected (*k*_*b*_ = 0, *k*_*p*_ = 1) (C) lines over the course of the passaging experiment. Points represent 12 independently evolving lineages per treatment, and curves show best-fit regression (adjusted *R*^2^ = 0.92, Table S1). All reported ODs are corrected for the OD of the blank medium.

Maintaining a small biofilm presence therefore has a relatively low cost, even in the absence of environmental stresses that favor biofilm cells, suggesting that selection against low rates of biofilm production will be weak. To test this hypothesis, we performed a selection experiment in which 12 replicate populations of *P. aeruginosa* were subjected to 20 transfers (approximately 130 generations), allowing only planktonic or only biofilm cells to survive (Fig. 4). While relative biofilm production rapidly decreased in the plankton-selected lines (*k*_*b*_ = 0, *k*_*p*_ = 1, red points in Fig. 4A), declining from ~40% to ~13% of the cells in biofilms after 7 transfers (paired *t* test, mean difference = 0.265, *t* = 18.7, df = 11, *P* = 1.1 × 10^−9^), biofilm production did not evolve to be any lower over the remaining 13 transfers of the experiment {Fig. 4A, biofilm fraction from passages 7 to 20 best fit by an intercept model [Akaike information criterion (AIC) = −580] versus a linear model [AIC = −424]; also note that in Fig. 4C, the biofilm optical density [OD] stays roughly constant over the course of the experiment}.

Interestingly, in the biofilm-only selection line (*k*_*b*_ = 1, *k*_*p*_ = 0; Fig. 4A, blue points), the proportion of cells in biofilms did not increase over the course of the experiment, although the total number of cells in the biofilm increased by more than 100% (Fig. 4B). Particularly, we see that the planktonic cell density increases steadily while the biofilm fraction increases initially and then stagnates after roughly 10 passages (Fig. 4B; Table S1). We did not observe significant morphological or phenotypic diversification in these lines (Fig. S3), suggesting that evolution of planktonic or biofilm growth-specialized lineages (15, 16) was not a major driver of population dynamics. This result is consistent with our models above, with planktonic growth driving biofilm accumulation, and experimentally demonstrates the utility of a mixed strategy under strict selection regimes.

10.1128/mBio.00672-17.7 TABLE S1  Regression model for passaging experiment. Parameter estimates for model fit in Fig. 4 are shown. Download TABLE S1, PDF file, 0.4 MB. Copyright © 2017 Lowery et al. 2017 Lowery et al. This content is distributed under the terms of the Creative Commons Attribution 4.0 International license.

10.1128/mBio.00672-17.6 FIG S3  Evolved populations did not significantly diversify. (Top) Three of the biofilm-selected lines were plated after 20 passages (roughly 130 generations) to assess the extent of diversification within each lineage. A single small-colony variant was detected in one of the lines (left, enlarged in the inset), and no variants were detected in the other two (not shown). These findings reflect the morphological diversity observed in the ancestral PAO1 line (right). (Bottom) Single clones from the ancestor and each of three populations under biofilm and planktonic selection were assayed for biofilm allocation (as in Fig. 4A), with *n* = 24 for lines biofilm.1 (the line from which the evolved photo was taken) and plankton.3 and *n* = 8 in all other cases. Levene’s test found no significant differences in variance between populations (biofilm selection, *P* = 0.81, *F* = 0.22 on 2 and 37 df; planktonic selection, *P* = 0.32, *F* = 1.15 on 2 and 37 df). Download FIG S3, PDF file, 0.1 MB. Copyright © 2017 Lowery et al. 2017 Lowery et al. This content is distributed under the terms of the Creative Commons Attribution 4.0 International license.

**FIG 5:**
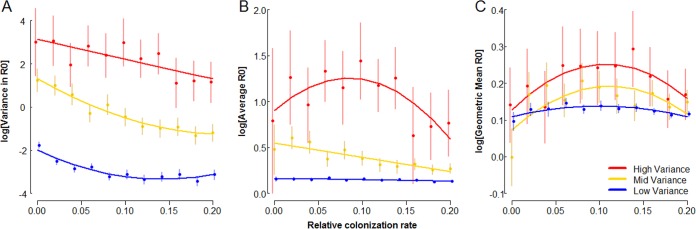
Biofilm production can act as a bet-hedging strategy. Shown are log-scale plots of variance in R0 (A), average R0 (B), and geometric mean R0 (C) as a function of the relative colonization rate (ratio of *c* to the expected value of *r*; see below) for lineages subjected to passaging simulations with different levels of environmental instability. One hundred replicate inocula of 5,000 planktonic cells with the same fixed colonization rates, *c* ∈ (0, 0.2), were subjected to 10 passages. In all cases, μ_*r*_ = 0.06, μ_*t*_ = 40, µ_*kp*_ = 0.1, and µ_*kb*_ = 0.6. Under fixed conditions, these parameters would favor a c* of 0.49. Variance treatments modified the σ^2^ of the distributions from which *r*, *t*, *k*_*p*_, and *k*_*b*_ were drawn, with σ^2^_low_ = µ/100, σ^2^_mid_ = µ/9, and σ^2^_high_ = µ/2.75. Parameters were restricted to logical values, with 0 ≤ *k*_*b*_, *k*_*p*_ ≤ 1; *r* > 0, and *t* ≥ 1. This thresholding did not change the overall mean of any parameter by more than ±3%. R0 was calculated as the ratio of founding cells at a given passage relative to the founding cells of the previous passage. Points and error bars (offset to reduce overlap) represent mean values and 95% confidence intervals, and solid lines display best-fit regressions (Table S2).

The low cost of maintaining a biofilm makes it well suited as a potential bet-hedging strategy, increasing the long-term geometric mean fitness in unpredictable environments by minimizing the variance in fitness through time. To test this prediction, we used our model framework to construct a simulated passaging regime in which growth and transmission parameters were subjected to differing levels of variance. We assigned inoculum populations of planktonic cells a fixed colonization rate, *c* (applied relative to *r*), and then subjected them to alternating periods of growth and transmission. The parameters *r*, *t*, *k*_*p*_, and *k*_*b*_ were drawn from normal distributions with fixed means µ and differing variances σ^2^ between treatments. The values of *r*, *t*, and *c* were used to solve equations 3 and 4; proportions *k*_*p*_ and *k*_*b*_ of these new cells were then passaged to the next growth phase, as in equation 7. The fitness function defined in equation 8 (effectively a reproductive number, R0 [14]) was used as our fitness metric for these simulations, calculated as the ratio of founding cells in a given passage relative to the number of founding cells in the previous passage. Simulation results are displayed in Fig. 5.

For all treatments, the variance in R0 declines with increasing *c*, consistent with biofilm colonization functioning as a bet-hedging strategy for bacteria facing unpredictable selection (Fig. 5A). For low and medium degrees of unpredictability, the average R0 declines monotonically with increasing *c*, reflecting the penalty imposed on fitness by environmental variance (Fig. 5B). Interestingly, under the high-variance treatment, a pronounced hump shape in the average R0 appears (Table S2), indicating that in extremely unpredictable environments, the trade-off between mean fitness and reduced variance in fitness breaks down and biofilm formation is generally beneficial (Fig. 5B). The combination of direct fitness benefits and bet hedging effects lead to maxima in geometric mean R0 at intermediate colonization rates across all variance treatments (Fig. 5C; Table S2).

10.1128/mBio.00672-17.8 TABLE S2  Regression models for bet-hedging simulations. Parameter estimates for models displayed in Fig. 5 are shown. Linear and quadratic regressions were fitted and compared for each variance treatment, and the best model was chosen on the basis of a lower AIC (47). Download TABLE S2, PDF file, 0.4 MB. Copyright © 2017 Lowery et al. 2017 Lowery et al. This content is distributed under the terms of the Creative Commons Attribution 4.0 International license.

## DISCUSSION

Many groups have use agent-based models to examine the processes and dynamics of biofilm formation (13, 17–20). While these studies benefit from explicit mechanistic foundations, it is not always straightforward to determine the underlying processes influencing the simulated populations. In this work, we constructed an analytical metapopulation model of allocation between coupled biofilm and planktonic compartments within a growing bacterial population, which allowed us to investigate the underlying population dynamics in detail. Within the biofilm, cell division limited by geometry and nutrient diffusion rendered its effects inconsequential to the dynamics of the population as a whole, relative to the exponentially expanding pool of planktonic cells. Under inhospitable conditions, in which the population experienced restricted growth and/or frequent disruption, trade-offs between the biofilm and planktonic compartments forced lineages to specialize in whichever fraction was favored for transmission between patches. When conditions become more permissive, the lineage is able to leverage exponential planktonic growth to maintain robust populations in both compartments and at a fraction of the cellular cost of direct biofilm allocation (i.e., at reduced colonization rates *c*); in general, such cost-saving measures are likely to be favored in any cooperative trait during periods of growth (21). Because maintenance of a biofilm comes at little cost under conditions favoring planktonic growth, biofilm is able to function as a robust and cost-effective hedge against unpredictable environmental change. Conversely, the planktonic fraction is a useful amplifier of biofilm cells even when biofilm is the transmissible propagule—this growth/transmission division of labor is more obvious for strictly nongrowing phenotypes (e.g., spores), but the same logic holds for slow growth in biofilms as well.

We focus primarily on biofilm-planktonic cell populations as a model of survival-fecundity alternate states and therefore use the language of colonization (of biofilms by planktonic cells) and dispersal (from the biofilm) to represent the processes of switching between the two phenotypes. However, the model logic applies to other classes of resistant cells as well, such as persisters and spores. Indeed, there are instances where biofilm formation functions as a prerequisite or amplifying step in the formation of other types of resistant cells; persister cells are often enriched in biofilms (8, 22–25), and biofilm formation is a prerequisite step in fruiting body formation (the preferred site of sporulation) in *Bacillus subtilis* (26, 27); it would be interesting to investigate how investment would be optimized with multiple survival phenotypes available in both simultaneous and sequential contexts. However, the generality of our models with respect to the specifics of a resistant cell type comes at the expense of mechanistic understanding. For example, we assume that resistant cells are continuously produced by vegetative cells; while this mechanism holds for persister cells, which by definition have no or extremely limited growth, it may not hold for established biofilms, where subsequent colonization by clonal planktonic cells can be blocked by extracellular matrix components (28), though the generality of this effect is not well studied. In our models and experiments, we primarily consider nascent populations and high-disturbance regimes and therefore reason that such exclusion would not have major impacts on our results but concede that such effects are likely to play a larger role in the long-term dynamics of the system. Further work to elucidate the mechanistic details of biofilm growth and attachment in similar experimental contexts would help to improve our understanding of how the colonization rate relates to biofilm accumulation as a function of time and population density.

Given the costs inherent to cellular investment in a growth-limited state, one may expect lineages to evolve the ability to efficiently switch between biofilm and planktonic phenotypes, thereby optimizing fitness by reducing lag times and minimizing unnecessary death in the event of environmental disturbance, and indeed, such systems appear to be abundant in nature (29–34). However, we note that environmental sensing in this case would not supplant the need to maintain some level of biofilm as a hedge (Fig. 4 and 5) but rather enhance the efficiency of such maintenance; in environments where catastrophic disturbances occur even at very low frequency, lineages that maintain biofilm regardless will still have better chances of survival. One would therefore predict biofilm to be completely lost in only the most constant environments.

Another likely outcome of long-term selection in environments that provide distinct spatial niches is genetic and morphological diversification within the population, resulting in coexisting lineages specialized to a particular niche (15, 16, 35, 36) that may or may not display similar division of labor between types. Such diversity can evolve rapidly but generally relies on the systems remaining undisturbed for at least several days (15, 35, 36); we speculate that this is due to saturation of readily available niches and thus increased selective pressure toward exploitation of any remaining sites within the environment. In experimental systems involving repeated dilutions similar to those presented here (16), diversity was slower to emerge, with smooth-colony variants appearing within 150 generations and more rugose variants only after 300 to 400 generations. Given that our experiments involved slower growth, more frequent dilutions, and fewer total passages, it is perhaps unsurprising that diversity would be slower to emerge (Fig. S3). Our models and experiments therefore suggest that microbial populations can enjoy the benefits of dividing labor between phenotypes within a single genotype and indeed may be at an advantage when the environment changes unpredictably (Fig. 5).

Phenotypic regulation to further optimize allocation between biofilm and planktonic lifestyles would help expedite the evolution of rudimentary life cycles at the population level, alternating between a growing “soma” and dispersive “propagules” with distinct demographies associated with each phase. Under the formalism presented here, either the biofilm or the planktonic compartment alone, or some combination thereof, may serve as dispersing propagules. The historical archetype has generally held that the biofilm functions as the soma, with motile planktonic cells as dispersive propagules (37–39). More recently, Hammerschmidt et al. (40) found that alternating selection on dispersive and biofilm phenotypes in *Pseudomonas fluorescens* leads to the evolution of a lifestyle in which cooperative biofilm cells producing shared adhesive molecules form a pellicle that functions as the growing soma, and planktonic nonadhesive cheats are coopted as dispersive propagules, thereby dividing labor between the two cellular fractions and increasing the overall fitness of the lineage. Our results indicate that the opposite cycle (biofilm cells as propagules, planktonic cells as soma) could also be viable, as the population can still reap the benefits of dividing labor between specialized cellular fractions. Indeed, where individual patches are permissive to growth but transmission between patches is exceedingly harsh (e.g., wind or animal dispersal), dispersal via biofilm aggregates and growth within a planktonic “soma” would offer the greatest advantage, as the “soma” would accumulate biomass rapidly, and dispersal propagules would enjoy increased survival at little reproductive cost given the hostile transmission conditions. For example, biofilm formation and other survival phenotypes are likely important to successful transmission via fomites, upon which bacteria can remain viable for months (41). Dispersal in physically linked groups (i.e., budding dispersal [42]) may also help maintain cooperative traits or competitive advantages (43), thereby potentially accelerating colonization when a new patch is reached. The biofilm “streamers” observed by Drescher et al. (44) may be another example of this mode of transmission, where flow rates are such that the biofilm forms physical bridges to allow colonization of vacant surfaces in a topographically complex environment, as full detachment would prevent recolonization of adjacent surfaces because of extreme shear forces.

Taken together, our results highlight the evolutionary significance of within-population phenotypic heterogeneity and its consequences for survival and fecundity in mixed-transmission environments. By optimizing the switching rate between robust and fecund specialists (here, the colonization rate from the planktonic to the biofilm fraction, though we note that other mechanisms could lead to equivalent outcomes, such as the steady-state frequencies of genotypes arising from within-population diversification, as in references 16 and 36), lineages were able to maximize fitness and transmission across a wide range of environments, as well as enhance survival in the face of catastrophic changes within the environment. The rate of phenotypic switching is therefore an essential parameter upon which selection may act when multiple phenotypes can persist within lineages.

## MATERIALS AND METHODS

### Passaging experiment.

A mid-exponential-phase culture of *P. aeruginosa* PAO1 was used to inoculate 200 µl of LB and one 3-mm sterile glass bead into each of 24 wells of a 96-well plate at an OD of 0.05. Plates were sealed with AeraSeal tape, and cultures were grown statically at 24°C in a humidified chamber. Every 12 h (growth conditions were chosen to prevent entry into stationary phase, where multiple regulatory systems that modulate biofilm and growth behaviors are engaged), biofilm allocation was measured by removing the liquid phase and measuring the OD, washing the attached biofilm with sterile water, resuspending it by vortexing it for 15 min in 200 µl of fresh LB, and then measuring the OD of the resuspended biofilm cells. Twelve lines had only the planktonic cells passaged, while the other 12 had only biofilm cells passaged; in each case, cells were diluted to an inoculum OD of 0.05 and 20 passages were performed. To assess whether morphological diversification has occurred under biofilm selection, aliquots from frozen stocks of three lineages were spread onto LB plates and morphology was assessed after incubation for 48 h at room temperature. Individual clones from each population were also selected and assayed for population allocation as described above.

### Statistics and mathematical analysis.

Agent-based simulations were performed with iDynoMiCs (13), and analyses were performed with Mathematica. Numerical modeling (45) and statistics were performed in R (46), unless otherwise noted.

## References

[1] Stearns SC 1992 The evolution of life histories. Oxford University Press, Bethesda, MD.

[2] De Paepe M, Taddei F 2006 Viruses’ life history: towards a mechanistic basis of a trade-off between survival and reproduction among phages. PLoS Biol 4:e193. doi:10.1371/journal.pbio.0040193. 16756387 PMC1475768

[3] Heineman RH, Brown SP 2012 Experimental evolution of a bacteriophage virus reveals the trajectory of adaptation across a fecundity/longevity trade-off. PLoS One 7:e46322. doi:10.1371/journal.pone.0046322. 23071555 PMC3470554

[4] Gudelj I, Weitz JS, Ferenci T, Claire Horner-Devine M, Marx CJ, Meyer JR, Forde SE 2010 An integrative approach to understanding microbial diversity: from intracellular mechanisms to community structure. Ecol Lett 13:1073–1084. doi:10.1111/j.1461-0248.2010.01507.x. 20576029 PMC3069490

[5] Green JL, Bohannan BJM, Whitaker RJ 2008 Microbial biogeography: from taxonomy to traits. Science 320:1039–1043. doi:10.1126/science.1153475. 18497288

[6] Cano RJ, Borucki MK 1995 Revival and identification of bacterial spores in 25- to 40-million-year-old Dominican amber. Science 268:1060–1064. doi:10.1126/science.7538699. 7538699

[7] Vreeland RH, Rosenzweig WD, Powers DW 2000 Isolation of a 250 million-year-old halotolerant bacterium from a primary salt crystal. Nature 407:897–900. doi:10.1038/35038060. 11057666

[8] Lewis K 2010 Persister cells. Annu Rev Microbiol 64:357–372. doi:10.1146/annurev.micro.112408.134306. 20528688

[9] Wood TK, Knabel SJ, Kwan BW 2013 Bacterial persister cell formation and dormancy. Appl Environ Microbiol 79:7116–7121. doi:10.1128/AEM.02636-13. 24038684 PMC3837759

[10] Van Acker H, Van Dijck P, Coenye T 2014 Molecular mechanisms of antimicrobial tolerance and resistance in bacterial and fungal biofilms. Trends Microbiol 22:326–333. doi:10.1016/j.tim.2014.02.001. 24598086

[11] Werner E, Roe F, Bugnicourt A, Franklin MJ, Heydorn A, Molin S, Pitts B, Stewart PS 2004 Stratified growth in *Pseudomonas aeruginosa* biofilms. Appl Environ Microbiol 70:6188–6196. doi:10.1128/AEM.70.10.6188-6196.2004. 15466566 PMC522130

[12] Fux CA, Costerton JW, Stewart PS, Stoodley P 2005 Survival strategies of infectious biofilms. Trends Microbiol 13:34–40. doi:10.1016/j.tim.2004.11.010. 15639630

[13] Lardon LA, Merkey BV, Martins S, Dötsch A, Picioreanu C, Kreft JU, Smets BF 2011 iDynoMiCS: next-generation individual-based modelling of biofilms. Environ Microbiol 13:2416–2434. doi:10.1111/j.1462-2920.2011.02414.x. 21410622

[14] Frank SA 1996 Models of parasite virulence. Q Rev Biol 71:37–78. doi:10.1086/419267. 8919665

[15] Rainey PB, Travisano M 1998 Adaptive radiation in a heterogeneous environment. Nature 394:69–72. doi:10.1038/27900. 9665128

[16] Poltak SR, Cooper VS 2011 Ecological succession in long-term experimentally evolved biofilms produces synergistic communities. ISME J 5:369–378. doi:10.1038/ismej.2010.136. 20811470 PMC3105725

[17] Hellweger FL, Clegg RJ, Clark JR, Plugge CM, Kreft JU 2016 Advancing microbial sciences by individual-based modelling. Nat Rev Microbiol 14:461–471. doi:10.1038/nrmicro.2016.62. 27265769

[18] Kreft JU 2004 Biofilms promote altruism. Microbiology 150:2751–2760. doi:10.1099/mic.0.26829-0. 15289571

[19] Momeni B, Waite AJ, Shou W 2013 Spatial self-organization favors heterotypic cooperation over cheating. eLife 2:e00960. doi:10.7554/eLife.00960. 24220506 PMC3823188

[20] van Gestel J, Nowak MA 2016 Phenotypic heterogeneity and the evolution of bacterial life cycles. PLoS Comput Biol 12:e1004764. doi:10.1371/journal.pcbi.1004764. 26894881 PMC4760940

[21] Cornforth DM, Sumpter DJT, Brown SP, Brännström Å 2012 Synergy and group size in microbial cooperation. Am Nat 180:296–305. doi:10.1086/667193. 22854073 PMC3635123

[22] Singh R, Ray P, Das A, Sharma M 2009 Role of persisters and small-colony variants in antibiotic resistance of planktonic and biofilm-associated Staphylococcus aureus: an in vitro study. J Med Microbiol 58:1067–1073. doi:10.1099/jmm.0.009720-0. 19528167

[23] Nadell CD, Xavier JB, Foster KR 2009 The sociobiology of biofilms. FEMS Microbiol Rev 33:206–224. doi:10.1111/j.1574-6976.2008.00150.x. 19067751

[24] Haaber J, Cohn MT, Frees D, Andersen TJ, Ingmer H 2012 Planktonic aggregates of Staphylococcus aureus protect against common antibiotics. PLoS One 7:e41075. doi:10.1371/journal.pone.0041075. 22815921 PMC3399816

[25] Høiby N, Bjarnsholt T, Givskov M, Molin S, Ciofu O 2010 Antibiotic resistance of bacterial biofilms. Int J Antimicrob Agents 35:322–332. doi:10.1016/j.ijantimicag.2009.12.011. 20149602

[26] Branda SS, González-Pastor JE, Ben-Yehuda S, Losick R, Kolter R 2001 Fruiting body formation by Bacillus subtilis. Proc Natl Acad Sci U S A 98:11621–11626. doi:10.1073/pnas.191384198. 11572999 PMC58779

[27] Aguilar C, Vlamakis H, Guzman A, Losick R, Kolter R, Eichenberger P 2010 KinD is a checkpoint protein linking spore formation to extracellular matrix production in Bacillus subtilis. mBio 1:e00035-10. doi:10.1128/mBio.00035-10. 20689749 PMC2912670

[28] Nadell CD, Drescher K, Wingreen NS, Bassler BL 2015 Extracellular matrix structure governs invasion resistance in bacterial biofilms. ISME J 9:1700–1709. doi:10.1038/ismej.2014.246. 25603396 PMC4511925

[29] Schuster M, Sexton DJ, Diggle SP, Greenberg EP 2013 Acyl-homoserine lactone quorum sensing: from evolution to application. Annu Rev Microbiol 67:43–63. doi:10.1146/annurev-micro-092412-155635. 23682605

[30] Cornforth DM, Popat R, McNally L, Gurney J, Scott-Phillips TC, Ivens A, Diggle SP, Brown SP 2014 Combinatorial quorum sensing allows bacteria to resolve their social and physical environment. Proc Natl Acad Sci U S A 111:4280–4284. doi:10.1073/pnas.1319175111. 24594597 PMC3964068

[31] Barraud N, Schleheck D, Klebensberger J, Webb JS, Hassett DJ, Rice SA, Kjelleberg S 2009 Nitric oxide signaling in *Pseudomonas aeruginosa* biofilms mediates phosphodiesterase activity, decreased cyclic di-GMP levels, and enhanced dispersal. J Bacteriol 191:7333–7342. doi:10.1128/JB.00975-09. 19801410 PMC2786556

[32] Huynh TT, McDougald D, Klebensberger J, Al Qarni B, Barraud N, Rice SA, Kjelleberg S, Schleheck D 2012 Glucose starvation-induced dispersal of Pseudomonas aeruginosa biofilms is cAMP and energy dependent. PLoS One 7:e42874. doi:10.1371/journal.pone.0042874. 22905180 PMC3419228

[33] Dow JM, Crossman L, Findlay K, He YQ, Feng JX, Tang JL 2003 Biofilm dispersal in Xanthomonas campestris is controlled by cell-cell signaling and is required for full virulence to plants. Proc Natl Acad Sci U S A 100:10995–11000. doi:10.1073/pnas.1833360100. 12960398 PMC196915

[34] Sauer K, Cullen MC, Rickard AH, Zeef LA, Davies DG, Gilbert P 2004 Characterization of nutrient-induced dispersion in *Pseudomonas aeruginosa* PAO1 Biofilm. J Bacteriol 186:7312–7326. doi:10.1128/JB.186.21.7312-7326.2004. 15489443 PMC523207

[35] Boles BR, Thoendel M, Singh PK 2004 Self-generated diversity produces “insurance effects” in biofilm communities. Proc Natl Acad Sci U S A 101:16630–16635. doi:10.1073/pnas.0407460101. 15546998 PMC528905

[36] Kim W, Levy SB, Foster KR 2016 Rapid radiation in bacteria leads to a division of labour. Nat Commun 7:10508. doi:10.1038/ncomms10508. 26852925 PMC4748119

[37] O’Toole GA, Kaplan HB, Kolter R 2000 Biofilm formation as microbial development. Annu Rev Microbiol 54:49–79. doi:10.1146/annurev.micro.54.1.49. 11018124

[38] Sauer K, Camper AK, Ehrlich GD, Costerton JW, Davies DG 2002 *Pseudomonas aeruginosa* displays multiple phenotypes during development as a biofilm. J Bacteriol 184:1140–1154. doi:10.1128/jb.184.4.1140-1154.2002. 11807075 PMC134825

[39] Monds RD, O’Toole GA 2009 The developmental model of microbial biofilms: ten years of a paradigm up for review. Trends Microbiol 17:73–87. doi:10.1016/j.tim.2008.11.001. 19162483

[40] Hammerschmidt K, Rose CJ, Kerr B, Rainey PB 2014 Life cycles, fitness decoupling and the evolution of multicellularity. Nature 515:75–79. doi:10.1038/nature13884. 25373677

[41] Kramer A, Schwebke I, Kampf G 2006 How long do nosocomial pathogens persist on inanimate surfaces? A systematic review. BMC Infect Dis 6:130. doi:10.1186/1471-2334-6-130. 16914034 PMC1564025

[42] Kümmerli R, Gardner A, West SA, Griffin AS 2009 Limited dispersal, budding dispersal, and cooperation: an experimental study. Evolution 63:939–949. doi:10.1111/j.1558-5646.2008.00548.x. 19154373

[43] Kragh KN, Hutchison JB, Melaugh G, Rodesney C, Roberts AEL, Irie Y, Jensen PØ, Diggle SP, Allen RJ, Gordon V, Bjarnsholt T 2016 Role of multicellular aggregates in biofilm formation. mBio 7:e00237-16. doi:10.1128/mBio.00237-16. 27006463 PMC4807362

[44] Drescher K, Shen Y, Bassler BL, Stone HA 2013 Biofilm streamers cause catastrophic disruption of flow with consequences for environmental and medical systems. Proc Natl Acad Sci U S A 110:4345–4350. doi:10.1073/pnas.1300321110. 23401501 PMC3600445

[45] Soetaert K, Petzoldt T, Setzer RW 2010 Solving differential equations in R: package deSolve. J Stat Softw 33:1–25. http://www.academia.edu/13415757/Solving_differential_equations_in_R_Package_deSolve.20808728

[46] R Core Team 2015, R: a language and environment for statistical computing. R Foundation for Statistical Computing, Vienna, Austria.

[47] Akaike H 1981 Likelihood of a model and information criteria. J Econom 16:3–14. doi:10.1016/0304-4076(81)90071-3.

