# Supplementary material for: Division of Labor, Bet Hedging, and the Evolution of Mixed Biofilm Investment Strategies
Source: mBio. 2017 Aug 8;8(4):e00672-17. doi: 10.1128/mBio.00672-17 (PMC5550747; doi:10.1128/mBio.00672-17)
Supplement: TEXT S1 [file mbo004173415s1.pdf]

Supplemental Text 1

Geometry and biofilm growth

Assume only the outside/edge grows owing to nutrient limitation.

1-dimension – assume cells arranged as a line segment.

$$\frac{dB}{dt} = 2r$$

as both ends of the line segment grow. This gives

$$B(t) = B_0 + 2rt$$

giving linear growth.

2-dimensions – assume cells arranged as a circle.

Growth depends on circumference. Assume cells have unit area. The relationship between cell number and circumference is then

$$C = 2\pi\sqrt{\frac{B}{\pi}}$$

So the dynamics are given by

$$\frac{dB}{dt} = rC = r2\pi\sqrt{\frac{B}{\pi}}$$

This gives

$$B(t) = B_0 + r2\pi\sqrt{\pi B}t + r^2\pi t^2$$

So growth is quadratic with time.

3-dimensions – assume cells arranged as a sphere.

Growth depends on surface area. Assume cells have unit area. The relationship between cell number and surface area is then

$$A = 4\pi\frac{B^{1/3}\left(\frac{3}{\pi}\right)^{1/3}}{2^{2/3}}$$

So the dynamics are given by

$$\frac{dB}{dt} = rA = r4\pi\frac{B^{1/3}\left(\frac{3}{\pi}\right)^{1/3}}{2^{2/3}}$$

This gives

$$B(t) = B_0 + r6^{2/3}\pi^{1/3}{B_0}^{2/3}t + 2\mathrm{x}6^{1/3}r^2\pi^{2/3}{B_0}^{1/3}t^2 + \frac{4}{3}r^3\pi t^3$$

So growth is cubic in time.
