# Supplementary figures and images for: Division of Labor, Bet Hedging, and the Evolution of Mixed Biofilm Investment Strategies
Source: mBio. 2017 Aug 8;8(4):e00672-17. doi: 10.1128/mBio.00672-17 (PMC5550747; doi:10.1128/mBio.00672-17)

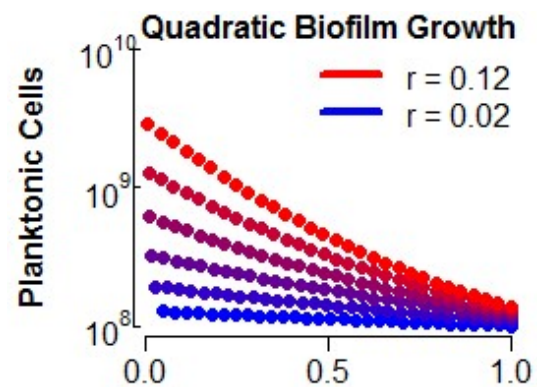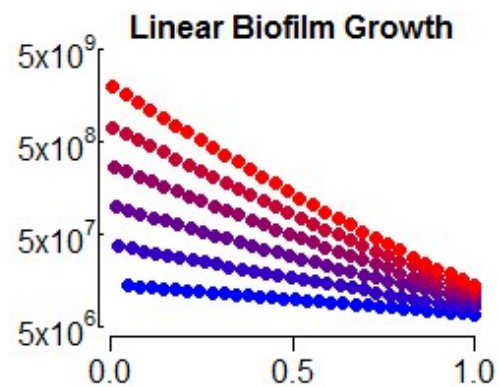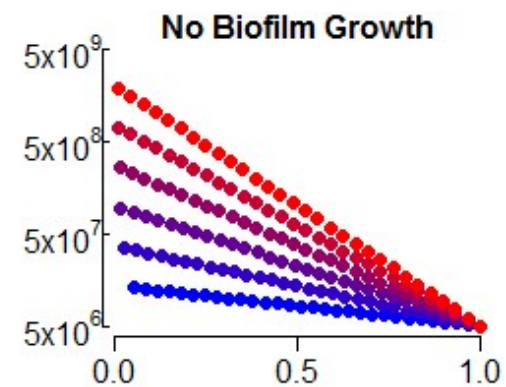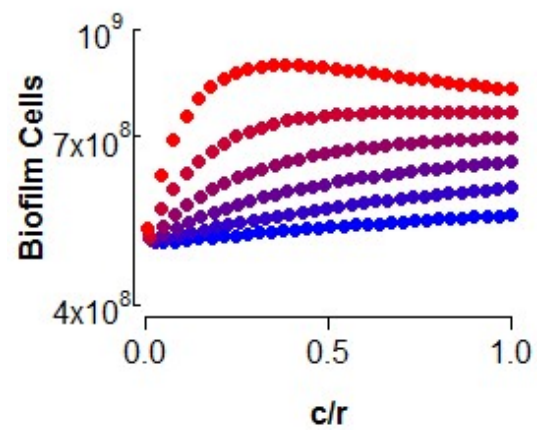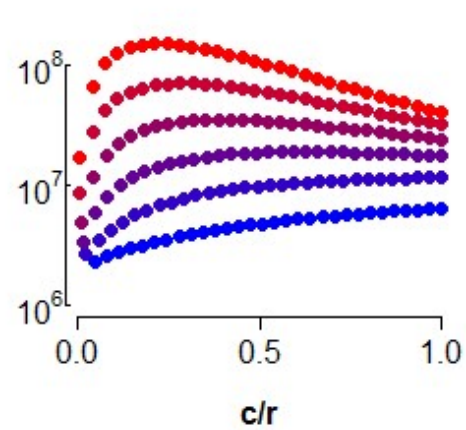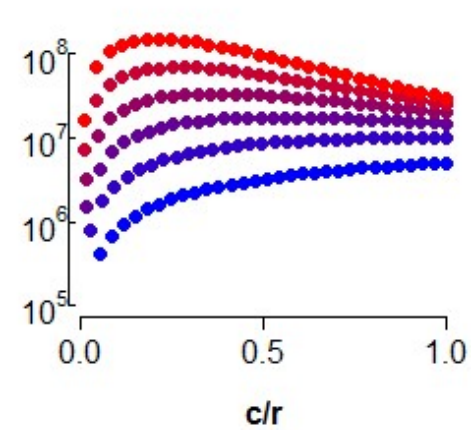

Supplement: FIG S1 [file mbo004173415sf1.pdf]

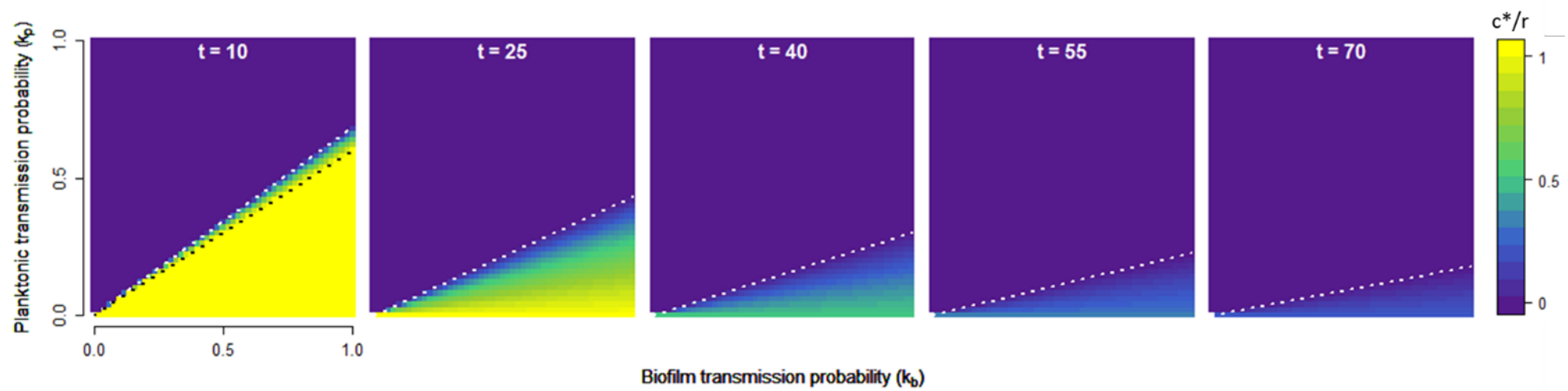

Supplement: FIG S2 [file mbo004173415sf2.pdf]

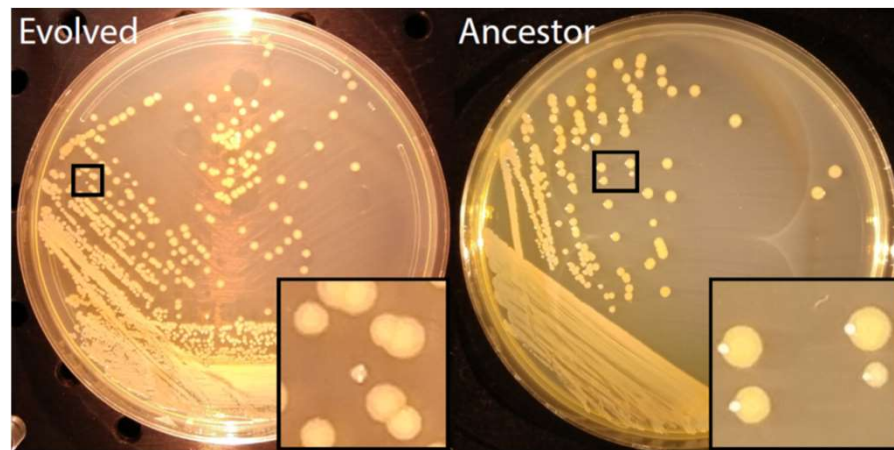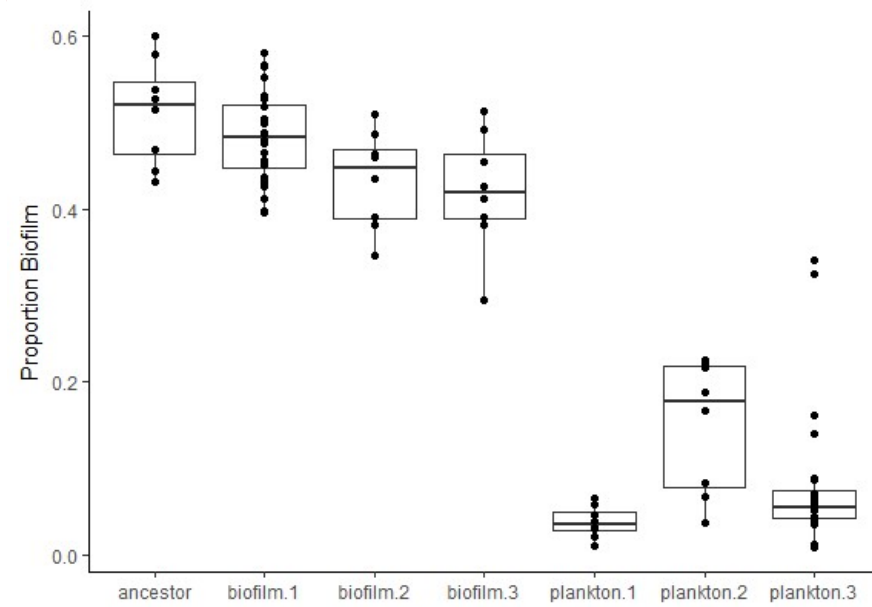

Supplement: FIG S3 [file mbo004173415sf3.pdf]
