# Supplementary material for: Division of Labor, Bet Hedging, and the Evolution of Mixed Biofilm Investment Strategies
Source: mBio. 2017 Aug 8;8(4):e00672-17. doi: 10.1128/mBio.00672-17 (PMC5550747; doi:10.1128/mBio.00672-17)
Supplement: TEXT S2 [file mbo004173415s2.pdf]

Supplemental Text 2

Derivation for Equation 2.3

Combining equations 2.1 and 2.2 yields

$$B(c,r,t) = \frac{c}{r-c} P_0(e^{(r-c)t} - 1)$$

Taking the derivative with respect to c, applying the quotient rule yields

$$\frac{dB}{dc} = \frac{P_0(r-c)\frac{d}{dc}\left[c\left(e^{(r-c)t} - 1\right)\right] - \frac{d}{dc}[r-c]ce^{(r-c)t} - 1)}{(r-c)^2}$$

Further evaluation gives

$$\frac{dB}{dc} = P_0(\frac{(r-c)\left(-tce^{(r-c)t} + e^{(r-c)t} - 1\right) + c(e^{(r-c)t} - 1)}{(r-c)^2}$$

which simplifies to

$$\frac{dB}{dc} = \frac{P_0((tc^2 - rtc + r)e^{(r-c)t} - r)}{(c-r)^2}$$

Taking the limit as c approaches r yields equation 2.3

$$\lim_{c \to r}\left(\frac{dB}{dc}\right) = -\frac{1}{2}P_0 t(rt - 2)$$
