# Supplementary material for: Division of Labor, Bet Hedging, and the Evolution of Mixed Biofilm Investment Strategies
Source: mBio. 2017 Aug 8;8(4):e00672-17. doi: 10.1128/mBio.00672-17 (PMC5550747; doi:10.1128/mBio.00672-17)
Supplement: TEXT S3 [file mbo004173415s3.pdf]

Supplemental Text 3

Evolutionary model - biofilm only / coexistence threshold

To examine the case where biofilm is maximally favored, we follow a similar logic to that employed in the analysis of the within-patch model in Equation 2.3 by examining the slope of W as c approaches r:

Our fitness function is defined as

$$W(c,r,t) = k_p e^{(r-c)t} + k_b(e^{(r-c)t} - 1)\frac{c}{r-c}$$

in Equation 3.2. Differentiating the first and second terms with respect to c gives

$$\frac{dW}{dc} = -k_p t e^{(r-c)t} + k_b \frac{(r-c)\frac{d}{dc}\left[c\left(e^{(r-c)t} - 1\right)\right] - \frac{d}{dc}[r-c]c(e^{(r-c)t} - 1)}{(r-c)^2}$$

via the product and quotient rules; further simplification yields

$$\frac{dW}{dc} = -k_p t e^{(r-c)t} + k_b \frac{(r-c)\left(-tce^{(r-c)t} + e^{(r-c)t} - 1\right) + c(e^{(r-c)t} - 1)}{(r-c)^2}$$

which simplifies to

$$\frac{dW}{dc} = -\frac{\left((k_b - k_p)tc^2 + (k_b - 2k_p)rtc + k_p r^2 t - k_b r\right)e^{(r-c)t} - k_b r}{(c-r)^2}$$

Evaluating the derivative as c approaches r yields equation 3.3,

3.3) $\lim_{c \to r}\left(\frac{dW}{dc}\right) = -\frac{1}{2}t\left(2k_p + k_b(rt-2)\right)$

In the limiting case of $k_p = 0$, we recover an equivalent scenario to that in 2.3, where biofilm (and thereby fitness) increases as c moves away from the minimum at zero, and is maximized at $c^* = r$ for $rt < 2$ (i.e. $\frac{dW}{dc}$ approaches the limit from below). Generally, fitness is maximized at $c^* = r$ when $\frac{k_p}{k_b} < \frac{rt-2}{2}$, and at $c^* < r$ when $\frac{k_p}{k_b} > \frac{rt-2}{2}$, setting the condition separating the strict trade-off and coexistence fitness regimes (black dashed line, Figure 3).

Evolutionary model, coexistence / plankton only threshold

Sole investment into the planktonic phase will be favored when fitness is maximized at $c^* = 0$; for our model, this condition is met when 1) $W(c=0) > \lim_{c \to r}(W)$, i.e. fitness declines with increasing c, and 2) $\frac{dW}{dc}(c=0) < 0$, i.e. the decline in fitness is monotonic. Evaluating Equation 3.2 under condition 1 yields

3.4) $k_p e^{rt} > k_p + k_b rt,$

while condition 2 gives

$\frac{k_b}{r}(e^{rt}-1) - \ k_p t e^{rt} < 0,$

which simplifies to

3.5) $k_p e^{rt} > \frac{k_b}{rt}(e^{rt}-1).$

Both conditions are linear in k_p and k_b; solving for k_p as a function of k_b in equations 3.4 and 3.5 and comparing the slopes reveals that Equation 3.5 presents the higher threshold for all values of *r* and *t* examined in this work. Therefore, we conclude cellular investment is routed wholly into the planktonic phase for $\frac{k_p}{k_b} > \frac{e^{rt}-1}{rt\ ^{rt}}$, while cells begin to be allocated into the biofilm when $\frac{k_p}{k_b} < \frac{e^{rt}-1}{rt\ ^{rt}}$ (white dashed line, Figure 3).
