# Supplementary material for: Division of Labor, Bet Hedging, and the Evolution of Mixed Biofilm Investment Strategies
Source: mBio. 2017 Aug 8;8(4):e00672-17. doi: 10.1128/mBio.00672-17 (PMC5550747; doi:10.1128/mBio.00672-17)
Supplement: TABLE S1 [file mbo004173415st1.pdf]

Model call: OD ~ fraction + passage + passage^2 + passage:selection + passage:fraction + passage:selection:fraction + passage^2:selection + passage^2:fraction + passage^2:selection:fraction

**Predictor**	**Estimate**	**SE**	**p-value**
Intercept	5.12E-02	4.62E-03	2.00E-16
fraction*P*	-2.38E-02	6.53E-03	0.0003
passage	1.08E-02	1.11E-03	2.00E-16
passage^2	-3.22E-04	5.52E-05	7.59E-09
passage:selection*P*	-9.26E-03	9.29E-04	2.00E-16
passage:fraction*P*	-4.90E-04	1.58E-03	0.76
passage:selection*P* :fraction*P*	3.24E-02	1.31E-03	2.00E-16
passage^2:selection*P*	2.84E-04	5.85E-05	1.41E-06
passage^2:fraction*P*	2.08E-04	7.81E-05	0.008
passage^2:selection*P* :fraction*P*	-8.44E-04	8.28E-05	2.00E-16

**$y = a + bx + cx^2$**	**a**	**b**	**c**
B-selection, B od	5.12E-02	1.08E-02	-3.22E-04
B-selection, P od	2.74E-02	1.08E-02	-1.14E-04
P-selection, B od	5.12E-02	1.54E-03	-3.80E-05
P-selection, P od	2.74E-02	3.35E-02	-6.74E-04
**Adjusted R^2:**	0.92		
**Residuals:**	*Min*	-0.124	
	1Q	-0.016	
	Med	-0.001	
	3Q	0.017	
	Max	0.150	
