# Supplementary material for: Division of Labor, Bet Hedging, and the Evolution of Mixed Biofilm Investment Strategies
Source: mBio. 2017 Aug 8;8(4):e00672-17. doi: 10.1128/mBio.00672-17 (PMC5550747; doi:10.1128/mBio.00672-17)
Supplement: TABLE S2 [file mbo004173415st2.pdf]

	Treatment	Predictor	Estimate	SE	p-value
**log[Variance R0]**	*low*	*Intercept*	-1.99	0.08	2.00E-16
		c	-19.02	1.78	2.00E-16
		c^2	67.19	8.58	1.17E-14
	mid	*Intercept*	1.32	0.17	6.78E-15
		c	-23.67	3.90	1.75E-09
		c^2	54.04	18.78	0.004
	high	*Intercept*	3.13	0.31	2.00E-16
		c	-9.14	2.59	4.31E-04
**log[Average R0]**	*low*	*Intercept*	0.16	0.005	2.00E-16
		c	-0.14	0.04	3.55E-04
	mid	*Intercept*	0.55	0.03	2.00E-16
		c	-1.58	0.28	2.02E-08
	high	*Intercept*	0.90	0.17	2.89E-07
		c	8.46	4.06	0.037
		c^2	-50.00	19.50	0.011
**log[Geometric Mean R0]**	*low*	*Intercept*	0.11	0.006	2.00E-16
		c	0.59	0.13	6.32E-06
		c^2	-2.95	0.62	2.51E-06
	mid	*Intercept*	0.07	0.02	2.09E-04
		c	2.15	0.45	2.26E-06
		c^2	-9.64	2.18	1.03E-05
	high	*Intercept*	0.13	0.04	5.10E-04
		c	2.32	0.85	0.007
		c^2	-10.80	4.10	0.009
